# Environmental bacteriophages active on biofilms and planktonic forms of toxigenic *Vibrio cholerae*: Potential relevance in cholera epidemiology

**DOI:** 10.1371/journal.pone.0180838

**Published:** 2017-07-10

**Authors:** Iftekhar Bin Naser, M. Mozammel Hoque, Ahmed Abdullah, S. M. Nayeemul Bari, Amar N. Ghosh, Shah M. Faruque

**Affiliations:** 1 Laboratory Sciences and Services Division, International Centre for Diarrhoeal Disease Research, Bangladesh, Dhaka, Bangladesh; 2 National Institute of Cholera and Enteric Diseases, Beliaghata, Calcutta, India; Universidade de Aveiro, PORTUGAL

## Abstract

In cholera-endemic areas, toxigenic *Vibrio cholerae* persist in the aquatic ecosystem mostly in a biofilm-associated state in which the bacteria remain embedded in an exopolysaccharide matrix. The biofilm-associated cells often enter into a dormant form referred to as conditionally viable environmental cells (CVEC), which resist cultivation on routine bacteriological media. However, these cells can naturally resuscitate into the active planktonic form through various mechanisms, multiply, and cause epidemics of cholera. This study was conducted to study possible effects of environmental bacteriophages on the prevalence and distribution of the pathogen between the biofilm associated state, and the planktonic form.

**Methods:**

Phages isolated from environmental waters in Bangladesh were tested for their host specificity towards *V*. *cholerae* O1 and O139, and the ability to disperse *V*. *cholerae* biofilms formed in the laboratory. Representative phages were further characterized by electron microscopy and whole genome sequencing. Selected phages were then introduced in various combinations to biofilms of toxigenic *V*. *cholerae* added to samples of river water, and the dispersion of biofilms as well as the growth kinetics of *V*. *cholerae* and the phages were monitored.

**Results:**

A phage cocktail composed of three different phages isolated from surface waters in Bangladesh and designated as JSF7, JSF4, and JSF3 could significantly influence the distribution and concentration of the active planktonic form and biofilm associated form of toxigenic *V*. *cholerae* in water. While JSF7 showed a biofilm degrading activity and dispersed cells from both *V*. *cholerae* O1 and O139 derived biofilms thus increasing the concentration of planktonic *V*. *cholerae* in water, JSF4 and JSF3 showed strong bactericidal activity against *V*. *cholerae* O1 and O139 respectively. A mixture of all three phages could effectively reduce both biofilm-associated and planktonic *V*. *cholerae* in river water microcosms.

**Significance:**

Besides potential applicability in phage-mediated control of cholera, our results have relevance in appreciating possible intricate role of diverse environmental phages in the epidemiology of the disease, since both biofilms and phages influence the prevalence and infectivity of *V*. *cholerae* in a variety of ways.

## Introduction

Bacterial biofilms have been implicated as a source of infection and contamination in medical and industrial settings as well as in waterborne transmission of pathogens [1,2,3]. Biofilms are surface-associated communities of bacteria encased in a matrix of complex heterogeneous extracellular polymeric substances composed of polysaccharides, proteins, nucleic acids, and lipids [1, 4]. Toxigenic *Vibrio cholerae*, the causative agent of cholera epidemics reside in an aquatic ecosystem and infects humans to cause the deadly disease [5]. In the aquatic environment, *V*. *cholerae* mostly exist in a dormant form referred to as conditionally viable environmental cells (CVEC) which resist cultivation in standard bacteriological medium, but may be cultured using certain modified techniques [3]. CVEC are derived from biofilms and comprise clumps of cells embedded in a dense matrix of exopolysaccharides [6]. At times, these dormant cells naturally resuscitate into active planktonic cells, and the occurrence of cholera epidemics are known to coincide with increased concentration of the active form of toxigenic *V*. *cholerae* in environmental waters [7]. Multiple ecological factors including bacteriophages, and metabolic products of diverse microorganisms have been proposed to influence the concentration of culturable *V*. *cholerae* in water [8,9,10].

Pathogenic bacteria in biofilms are particularly difficult to eradicate because they exhibit resistance to antimicrobial treatments [11, 12], and often act as the source of a high dose of the pathogen. Thus, to devise cholera control measures, and to better understand the ecology of the pathogen, it is important to characterize agents that can influence the environmental prevalence of pathogenic *V*. *cholerae* as well as their distribution between the biofilm-associated and planktonic forms. In the present study, we characterized 3 different environmental phages which act on *V*. *cholerae* (vibriophage), and tested the effect of administering these phages on biofilm associated *V*. *cholerae* in laboratory microcosms. One of these phages could degrade biofilm matrix of *V*. *cholerae*, and increase the concentration of planktonic *V*. *cholerae* in water, whereas the other two phages could effectively kill planktonic *V*. *cholerae* O1 and O139 cells respectively. These results suggested possible cooperation of diverse phages in modulating the prevalence, and distribution of pathogenic *V*. *cholerae* in the aquatic ecosystem. Furthermore, the results of this study may contribute towards developing effective phage mediated treatment of water as a potential approach to reduce the risk of waterborne diseases, such as cholera.

## Materials and methods

### Environmental phages and *V*. *cholerae* host strains

*Vibrio cholerae* specific phages JSF3, JSF4 and JSF7 were originally isolated from environmental water samples in Bangladesh by soft agar plaque assays using different indicator *V*. *cholerae* strains available in our collection [8, 13]. Of a total of 36 vibriophages in our collection, JSF3 is a *V*. *cholerae* O139 specific phage, JSF4 represents one of 34 *V*. *cholerae* O1 El Tor biotype specific phages, whereas JSF7 phage is capable of degrading biofilms of both *V*. *cholerae* O1 and O139, but forms plaques only on *V*. *cholerae* O1 El Tor biotype strains. One or more of the indicator strains including G-3669 (El Tor), P-27457 (El Tor), AI 1852 (O139), and MO1220 (O139) were used as host bacteria for purification and amplification of different phages for the present study. Phages were stored in SM buffer (100 mM NaCl, 8.1 mM MgSO4, 0.05 mM Tris-Cl [pH 7.5], 0.01% gelatin) at 4^°^C.

### Plaque assay for quantification of phages

Logarithmic-phase cells (500 μl) of a host bacterial strain in nutrient broth (Difco, Detroit, Mitch.) were mixed with 3.5 ml aliquots of soft agar (nutrient broth containing 0.8% Bactoagar, Difco), and the mixture was overlaid on nutrient agar plates. Samples tested for the presence of phages including aliquots of water or bacterial culture supernatant (10–50 μl), were pre-filtered through 0.22μm pore size filters (Millipore Corporation, Bedford, MA) to make them bacteria-free, inoculated on the plates, and incubated for 16 h at 37^°^C. A sample was scored positive for phages when a plaque was observed on the bacterial lawn in the plates. Plaques were counted to estimate the concentration of phage particles in the sample.

### Phage-production and testing host specificity

A single discrete phage plaque was purified three times by the soft agar (0.7%) overlay method [14] with a susceptible *V*. *cholerae* strain. For growing the phage in liquid medium, an overnight culture of the host strain was diluted 1:100 in fresh nutrient broth and grown at 37^°^C for 4 h. The culture was then inoculated with phages from a single plaque. The bacterium-phage culture was incubated at 37^°^C for 16h, when lysis of most of the bacteria occurred. The culture was centrifuged at 10,000 x *g* for 20 min, and the supernatant was filtered through a 0.22 μm pore size filter (Millipore). The number of phage particles in the filtered supernatant was determined by testing serial dilutions of the supernatant by the soft agar overlay method with the propagating strain. The host range for the phage was tested at a titer of 10^3^ pfu/ml using a variety of bacterial strains (S1 Table).

### Stability of phages

The effects of temperature, pH, and salinity on the stability of the phages were assessed as described previously [15]. Briefly, a defined number of phage particles were added into SM buffer pre-adjusted to different conditions of salinity and pH, and were incubated at different temperatures. The titer of phage particles remaining after 6h was expressed as a percentage of the original titer.

### Preparation and estimation of biofilms

Biofilms were formed in borosilicate glass tubes as described previously [16]. Briefly, colonies of the appropriate *V*. *cholerae* strain grown on Luria-Bertani (LB) agar plates were resuspended in LB broth at an optical density of 0.6 at 600 nm. The suspension was diluted 100 folds in LB broth and was inoculated into multiple borosilicate glass tubes. Biofilms were formed by allowing these cultures to stand for 24h at room temperature. Quantification of biofilms was done by spectrophotometry using crystal violet stained biofilm cells as described previously [16, 17]. Briefly, tubes containing biofilms were rinsed vigorously with distilled water to remove non-adherent cells, filled with 1 ml of a 0.1% crystal violet solution (Sigma-Aldrich. Inc., St. Louis, USA), and allowed to stand for 30 min. The tubes were again rinsed three times with water to remove non-adherent dye. The cell associated dye was extracted with 1 ml of dimethyl sulfoxide (DMSO), and optical density of the suspension was measured at 570 nm, to estimate biofilms.

### Assay of biofilm-dispersing activity

To identify phages capable of dispersing biofilms of *V*. *cholerae*, biofilms prepared in the laboratory were exposed to different phages and examined for degradation of the biofilm matrix and release of free cells. Biofilms of the appropriate *V*. *cholerae* strains were established on the sides of a series of glass tubes as described above. Free cells were washed away and tubes with biofilms attached to the inner surfaces were retained. Biofilm was measured in one or more representative tubes by staining with crystal violet followed by dye-extraction and measurement of OD at 550 nm. Remaining tubes with the biofilms were inoculated with 1 ml LB broth containing 1.5 x 10^7^ pfu of JSF7 phage and held at room temperature. Control tubes were also inoculated with 1 ml LB broth but without the phage. At different time intervals, viable count of *V*. *cholerae* was measured in the aqueous phase. Biofilms retained in the tubes after exposure to the phage was also estimated using crystal violet-staining as described above. Biofilms derived from both phage sensitive and phage resistant strains were used in these assays.

### Monitoring of biofilm dispersion and *V*. *cholerae* concentration in microcosms

Biofilms were prepared as described previously [17] on 22 × 22 mm^2^ cover slips placed in petri dishes in which the relevant *V*. *cholerae* culture was added and maintained at room temperature in static condition for 24h. River water obtained from the Buriganga river in Dhaka city was sterilized by filtration through a series of filters including 0.22μM pore sized millipore filter. Cover slips were washed to remove free cells and a set of 20 cover slips were equally distributed in four 500 ml conical flasks containing 200 ml filtered river water. A mixture of three phages JSF3, JSF4 and JSF7 (~3.0 x 10^9^ pfu each) in 3 ml LB was added to each flask. The flasks were shaken at low speed (~50 rpm) at room temperature to ensure uniform contact of the cover slips to the solution in the flask. Aliquots of water samples were removed every 2 hours to estimate free *V*. *cholerae* cells, and a cover slip was removed to estimate residual biofilm using crystal vilolet-staining, extraction with DMSO followed by spectrophotometry at 550 nm as described above.

### Assessing phage resistance of biofilm-associated *V*. *cholerae*

*V*. *cholerae* biofilms were exposed to phages JSF3 and JSF4 which did not have biofilm-degrading activity, to assess the susceptibility of biofilm associated *V*. *cholerae* to these phages. Biofilms were prepared on a series of identical glass test tubes using the *V*. *cholerae* O1 El Tor strain C6706 or an O139 strain MO1220. Phage susceptibility of biofilm-associated cells were compared to that of planktonic cells in two different states. These included a fresh culture of the *V*. *cholerae* strain containing mostly planktonic bacteria, and suspension of cells dispersed from biofilms by physical agitation. To prepare biofilm-derived planktonic cells, biofilms were disrupted by shaking with glass beads and suspended in LB. Multiple aliquots of planktonic cell samples and intact biofilms were exposed to the lytic phage JSF3 or JSF4 at 1 x 10^3^ pfu/ml in LB supplemented with 5 mM CaCl_2_ and 5 mM MgCl_2_ at 37^°^C with shaking. Appropriate control assays without a phage or with a phage to which the target bacteria was resistant were run in parallel i.e., O139 specific phage JSF3 used with O1 strain C6706 and vice versa. The tubes with planktonic cells were removed at regular intervals, cells were precipitated by centrifugation, washed in fresh LB broth and dilutions were plated on LB agar plates to determine viable cell counts. For tubes with intact biofilms, cells were disrupted by shaking with glass beads, and the suspension was centrifuged to precipitate cells, which were washed in fresh LB broth, before plating. The apparent survival rate of *V*. *cholerae* cells after incubation for different time periods was calculated, and expressed as a percentage of the initial number of cells.

### Isolation and analysis of phage nucleic acids

For isolation and analysis of phage nucleic acids, culture supernatants containing phage particles were filtered through 0.22 μm pore-sized filters (Millipore). The filtrates were mixed with one-fourth volume of a solution containing 20% polyethylene glycol (PEG-6000) and 10% NaCl, and centrifuged at 12000 x *g* to precipitate phage particles. The precipitate was dissolved in a solution containing 20 mM Tris-Cl (pH 7.5), 60mM Kcl, 10mM MgCl, 10mM NaCl, and digested with pancreatic DNAseI (100 units/ml) and RNAse A (50 μg/ml) at 37^°^C for 2 hours. The solution was extracted with phenol-chloroform, and the total nucleic acids were precipitated with ethanol. Phage nucleic acids were suspended in deionized water and purified using the SV Minipreps DNA purification system (Promega Madison, USA). The phage nucleic acid was digested with restriction endonucleases (Invitrogen Corporation, Carlsbad, CA) and analyzed by agarose gel electrophoresis following standard procedures to initially check for diversity and select different phages for sequencing.

### Sequencing of phage genomes

The phage genomes were sequenced at the icddr,b core genomics facility using Illumina based technology. Genomic fragment libraries for whole-genome sequencing were prepared using Illumina Nextera® XT DNA library Preparation Kit (Cat. no, FC-131-1024) as per manufacturer's instructions, and sequencing was conducted with an Illumina MiSeq sequencer. The sequences were quality checked, assembled into contigs, and aligned with reference sequences using softwares available on-line at Illumina BaseSpace (https://basespace.illumina.com/lab).

### Electron microcopy of phage particles

A high titer phage preparation (~10^10^ pfu/ml) was obtained using the plate lysis procedure as described previously [18]. Five μl of the phage suspension was deposited on a carbon-coated copper grid and was allowed to adsorb for 1 min. The excess liquid was blotted out with a filter paper and phage particles were stained with 2% aqueous solution of uranyl acetate. Grids were examined with a FEI transmission electron microscope (model Tecnai 12 BioTwin). Length measurements were done using analySIS software (SIS GmbH, Germany).

### Statistical analysis

General statistical analysis of data was done using the in-built data analysis program in Microsoft Excel (MS office version 2007). Data were expressed as mean ± standard deviation, and differences were tested by two-tailed t-test. The values of P < 0.05 were considered statistically significant.

### Institutional approvals

All experimental protocols were approved by the Research Review Committee (RRC) and the Ethics Review Committee (ERC) of the icddr,b (Protocol numbers PR-15029 and PR-07018). All methods were conducted in accordance with the guidelines of the RRC and ERC.

## Results

### Phenotypic and genetic characteristics of JSF3, JSF4 and JSF7 phages

Phages JSF3, JSF4 and JSF7 were initially isolated from different samples of river water in Dhaka, Bangladesh. The host specificity of these phages was examined by using a panel of strains belonging to different species or serogroups (S1 Table). Only *V*. *cholerae* O1 strains were susceptible to JSF4 and JSF7 phages, whereas the JSF3 phage was specific for *V*. *cholerae* O139 strains. JSF7 was found to be capable of dispersing biofilms formed by both *V*. *cholerae* O1 and O139 strains. All three phages produced clear plaques with a diameter of ~ 1 mm on a lawn of their respective host bacteria.

**Fig 1:**
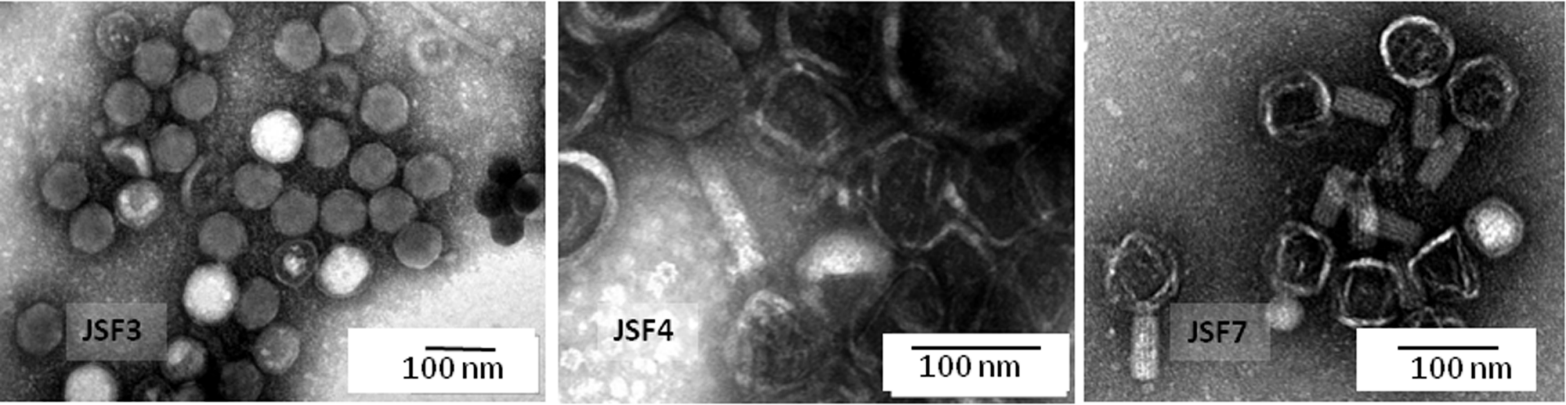
Electron micrograph showing the morphology of three different *V*. *cholerae* specific phages. Note the hexagonal head and tails of various lengths.

Electron microscopic examination revealed that all three phages had isomeric heads and while JSF3 had a short tail, JSF4 had a long non-contractile tail, and JSF7 had a contractile tail (Fig 1). Based on the morphology, JSF3 belonged to the family Podoviridae, whereas JSF4 belonged to Siphoviridae and JSF7 belonged to Myoviridae family [19]. A comparison of the characteristics of JSF3, JSF4 and JSF7 phages is presented in Table 1.

**Table 1:** Phenotypic and genetic characteristics of JSF3, JSF4 and JSF7 vibriophages.

Characteristics	Designation of Phages		
	JSF3	JSF4	JSF7
Morphology	Isometric head with short non-contractile tail	Isometric head with long non-contractile tail	Isometric head with long contractile tail
Family	*Podoviridae*	*Siphoviridae*	*Myoviridae*
Head diameter	58.3 ± 4.0	62.3 ± 2.5	58.3 ± 4.0
Tail length	10.8 ± 2.0	86.9 ± 3.3	55.9 ± 2.5
Tail width	-	15.5 ± 1.7	24.4 ± 0.8
Nucleic acid	Double stranded DNA	Double stranded DNA	Double stranded DNA
Genome size	69Kb	124Kb	46Kb
GC percentage	37.81	37.08	48.42
Host- specificity	*V*. *cholerae* O139	*V*. *cholerae* O1, El Tor	*V*. *cholerae* O1, El Tor
Dispersion of biofilms of *V*. *cholerae* O1 and O139	Negative	Negative	Positive

All three phages were stable at temperature below 37°C. The stability decreased with rise of temperature and more than 80% of phages were rapidly inactivated at temperatures above 45^°^C. The phages also remained mostly infectious (65% to 98%) at pH ranging from 6.0 to 9.0. (S1 Fig). Phage particles were stable and remained infectious for more than 4 weeks when they were stored at room temperature in SM buffer (100 mM NaCl, 8.1 mM MgSO4, 0.05 mM Tris-Cl [pH 7.5], 0.01% gelatin).

### Dispersion of biofilm associated *V*. *cholerae* cells by JSF7 Phage

**Fig 2:**
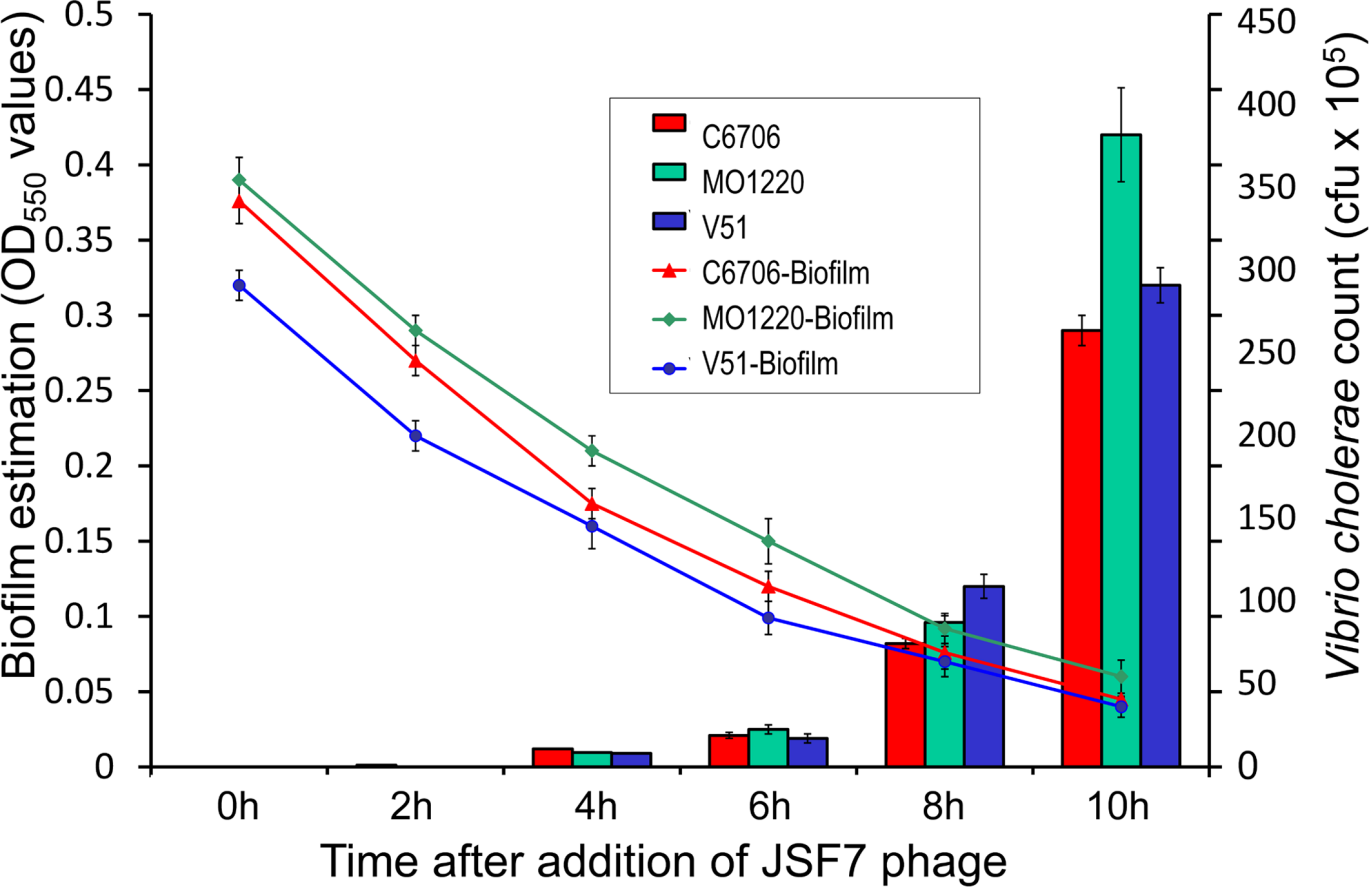
Degradation of biofilms by JSF7 phage and concomitant increase in corresponding *V*. *cholerae* count. Biofilms of the indicated strains were established on the sides of glass tubes by static incubation in LB medium. After washing away planktonic cells, the biofilm was measured by staining with crystal violet followed by dye-extraction and measurement of OD at 550nm. Viable count of *V*. *cholerae* cells in the aqueous phase was determined at the indicated times after addition of JSF7 phage (1.5 x 10^7^ pfu/ml). Note also that strains V51 and MO1220 are resistant to JSF7 phage, whereas strain C6706 is susceptible. Thus JSF7 phage degrades the biofilm matrix of both phage resistant and susceptible strains and releases planktonic cells. Each data-point represents the mean value and standard deviation of three independent observations.

The JSF7 phage was found to degrade biofilm matrices of both *V*. *cholerae* O1 and O139 and cause dispersion of the biofilm associated cells (Fig 2), suggesting that the process does not require infection of the bacterial cells by the phage. Addition of 1.5 x 10^7^ pfu of the phage into borosilicate vials with biofilms attached on the inner surface, caused noticeable biofilm degradation by 2h and reduced biofilms biomass by 50% within 6h. The dispersion of biofilms was associated with rise in free bacterial cell count in the medium (Fig 2). However, the observed cell count of the phage-susceptible strain C6706 in the aqueous phase was lower than those of the phage-resistant strains MO1220 and V51 used in this study, indicating that a proportion of the susceptible cells were presumably killed by the phage after their dispersion from the biofilm matrix.

### Phage resistance of biofilm-associated *V*. *cholerae*

**Fig 3:**
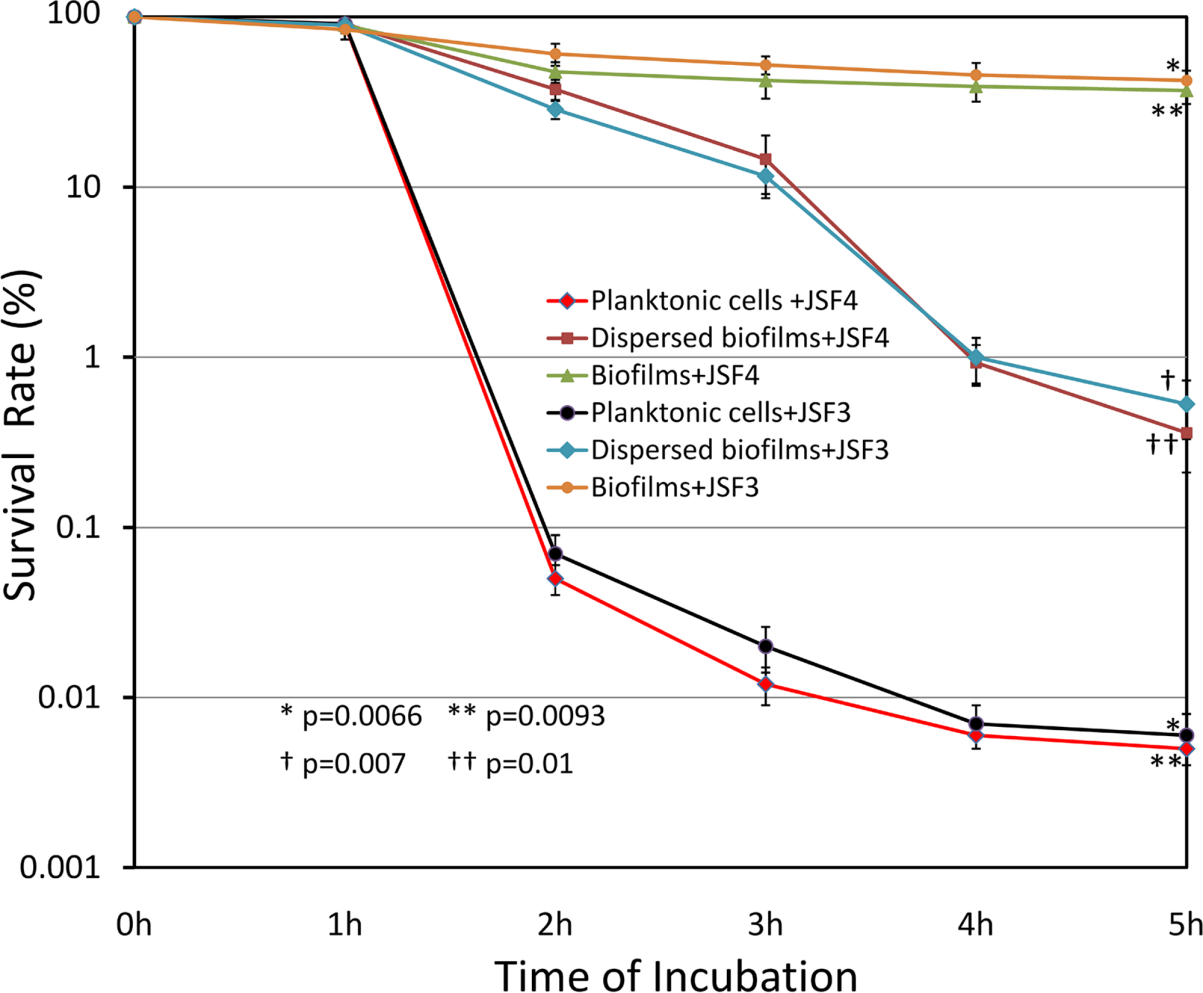
Resistance of biofilm-associated *Vibrio cholerae* to lytic phages JSF3 and JSF4. Phage susceptibility of biofilm-associated cells were compared to that of respective freshly grown planktonic cells or cells physically dispersed from biofilms by shaking with glass beads and suspended in LB. Different cell suspensions and intact biofilms were exposed to 1 x 10^3^ pfu/ml of JSF3 or JSF4 phage in LB supplemented with 5 mM CaCl_2_ and 5 mM MgCl_2_ and incubated at 37^°^C with shaking. At different time intervals, the residual bacterial cells were estimated. The survival rate of planktonic and biofilm-associated cells of *V*. *cholerae* O1 El Tor strain C6706 and O139 strain MO1220 after exposure to specific phages JSF4 and JSF3 respectively for different time periods are shown. Data points represent mean values and standard deviations of three different observations. The p values for the difference between survival of biofilm associated cells and planktonic cells when exposed to JSF3 (*) and JSF4 (**), and those for *V*. *cholerae* cells dissociated from biofilms by physical agitation vs fresh cultures of the bacteria exposed to JSF3 (†) and JSF4 (††), are shown.

Besides JSF7, the other two lytic phages included in this study, namely JSF3 and JSF4 were also tested for their activity on biofilms, but these two phages did not show any biofilm-degrading activity (data not shown). Phages JSF3 and JSF4 were also used to test the susceptibility of biofilm-associated *V*. *cholerae* to these phages, as compared to that of the planktonic form of the same bacteria. As shown in Fig 3, while the planktonic cells were killed by the phages at high rate, the biofilm-associated cells were mostly resistant to these lytic phages that do not have biofilm-degrading activity. The difference in survival rate between biofilm associated cells and corresponding planktonic cells was apparent after 2h of incubation with the phage, and after 5h of incubation the difference between the survival of biofilm associated cells as compared to planktonic cells was highly significant both for phage JSF4 (p = 0.0090) and for JSF3 (p = 0.0066). Remarkably, *V*. *cholerae* cells dissociated from biofilms by physical agitation maintained the enhanced resistance phenotype, and remained significantly more phage-resistant than fresh cultures of the bacteria (p = 0.01 for JSF4 and p = 0.007 for JSF3).

### Effect of phages on biofilm associated *V*. *cholerae* in river water

Three different phages, JSF3, JSF4 and JSF7 were inoculated in different combination as well as separately into flask containing pre-formed biofilms (on 22mm × 22 mm cover slips) and filtered river water. The pH range of the water was found to be 7.5 to 8.0, and the salinity was 0.4 to 0.5 parts per thousand (ppt). The flasks were incubated at room temperature, and viable cell count and phage counts in the aqueous phase were monitored. We found that phage JSF7 was able to disperse biofilm-associated cells and thus increase the concentration of planktonic cells in the aqueous phase (Table 2). For *V*. *cholerae* O139 strain MO1220, which was resistant to lysis by JSF7 phage this increase was more than 10^6^ fold in 6h, whereas for *V*. *cholerae* O1 strain C6706 which was susceptible to lysis by JSF7 the residual increase in cell count was about 75 folds. The increase in cell counts in the aqueous phase was consistent with the reduction in biofilm matrix retained on the cover slips, as assayed by crystal violet staining (Table 2). As mentioned above, the observed concentration of dispersed cells in the aqueous phase was significantly lower when the biofilm strain was susceptible to the phage as compared to a strain which was resistant (p = 0.0034), indicating that a proportion of the susceptible cells were killed by the phage after their dispersion from the biofilm matrix. This assumption was further supported by an observed rise in phage titer indicating amplification of the phage using susceptible host bacteria (Table 2). When biofilms of *V*. *cholerae* were exposed to JSF3 and JSF4 phages in the absence of JSF7 phage there was no significant change in the counts of *V*. *cholerae* cells in the aqueous phase or the density of the biofilm matrix. However when a combination of phages including JSF7 were used on biofilms of a phage susceptible *V*. *cholerae*, the biofilm strain released from the matrix were almost completely eliminated. For example, when biofilms of strain C6706 was treated with phage JSF4 and JSF7 together, only about 5 cfu/ml of the bacteria survived the phage treatment. When all three phages were used simultaneously to treat biofilms of either strain C6706 or MO1220, only a mean cfu of 2.7 x 10 to 3.2 x 10 cells/ml survived after 6h of incubation (Table 2). Similarly, very low survival rates of the bacteria were observed when mixed biofilms of *V*. *cholerae* O1 and O139 strains C6706 and MO1220 were exposed to all three phages (Table 2).

**Table 2:** Activity of diverse phages on biofilms of *V*. *cholerae* O1 or O139 in microcosms.

*V*. *cholerae* strains (serogroup) used to prepare biofilms	Phages used	Various parameters measured after 6h of incubation with or without adding phage				
		Colony count without phage (cfu/ml)	Colony count with phage (cfu/ml)	Phage count (pfu/ml)	Biofilm (OD_550_) without phage	Biofilm (OD_550_) with phage
C6706 (O1, El Tor)	JSF3	0.5 x 10^2^ ± 0.4 x 10	0.9 x 10^2^ ± 1.1 x 10	7.5 x 10^6^ ± 2.0 x 10^5^	0.45 ± 0.05	0.42 ± 0.05
MO1220 (O139)	JSF3	1.2 x 10^2^ ± 2.0 x 10	0.3 x 10^2^ ± 0.3 x 10	1.5 x 10^7^ ± 1.5 x 10^6^	0.51 ± 0.04	0.5 ± 0.05
C6706 (O1, El Tor)	JSF4	1.5 x 10^2^ ± 1.7 x 10	0.6 x 10^2^± 0.4 x 10	1.4 x 10^7^ ± 2.0 x 10^6^	0.41 ± 0.03	0.39 ± 0.04
MO1220 (O139)	JSF4	1.2 x 10 ± 1.5 x 10	1.5 x 10^2^ ± 1.7 x 10	1.0 x 10^7^ ± 5.0 x 10^5^	0.51 ± 0.08	0.48 ± 0.08
C6706 (O1, El Tor)	JSF7	0.75 x 10^2^ ± 0.4 x 10	5.2 x 10^3^ ± 6.6 x 10^2^	7.3 x 10^8^ ± 2.0 x 10^7^	0.48 ± 0.05	0.15 ± 0.02
		*Fold increase in cfu = 70.09 ± 12.69				
MO1220 (O139)	JSF7	1.2 x 10^2^ ± 1.7 x 10	7.5 x 10^8^ ± 6.2 x 10^7^	1.4 x 10^7^ ± 1.1 x 10^6^	0.55 ± 0.1	0.21 ± 0.03
		*Fold increase in cfu = 6298701 ± 650777				
C6706 (O1, El Tor)	JSF3, JSF4	1.5 x 10^2^ ± 0.5 x 10	0.9 x 10 ± 0.05 x 10	JSF3 1.1 x10^7^ ± 2.0 x10^6^ JSF4 8.2 x10^7^ ± 1.3 x10^7^	0.51 ± 0.02	0.48 ± 0.01
MO1220 (O139)	JSF3, JSF4	1.2 x 10^2^ ± 1.5 x 10	0.14 x 10^2^ ± 0.1 x 10	JSF3 9.0 x10^7^ ± 1.2 x10^7^ JSF4 1.2 x10^7^± 2.6 x10^6^	0.48 ± 0.03	0.47 ± 0.03
C6706 (O1, El Tor)	JSF3, JSF7	0.5 x 10^2^ ± 0.3 x 10	2.2 x 10^2^ ± 1.7 x 10	JSF3 1.2 x10^7^ ± 3.0 x10^6^ JSF7 6.2 x 10^8^ ± 1.1 x10^8^	0.48 ± 0.02	0.11 ± 0.02
MO1220 (O139)	JSF3, JSF7	8.1 x 10 ± 0.7 x 10	1.4 x 10^2^ ± 2.0 x 10	JSF3 5.4 x10^8^ ± 8.0 x10^7^ JSF7 1.1 x 10^7^ ± 1.1 x10^6^	0.46 ± 0.06	0.20 ± 0.01
C6706 (O1, El Tor)	JSF4, JSF7	0.5 x 10^2^ ± 0.3 x 10	0.5 x 10 ± 0.1 x10	JSF4 8.4 x10^7^ ± 8.0 x10^6^ JSF7 7.4 x 10^7^± 1.0 x10^7^	0.52 ± 0.05	0.12 ± 0.02
MO1220 (O139)	JSF4, JSF7	1.1 x 10^2^ ± 0.5 x 10	6.5 x 10^8^ ± 1.0 x 10^7^	JSF4 1.0 x10^7^ ± 1.5 x10^6^ JSF7 1.1 x 10^7^ ± 1.2 x10^6^	0.47 ± 0.04	0.22 ± 0.02
C6706 (El Tor)	JSF3, JSF4, JSF7	0.5 x 10^2^ ± 0.3 x 10	0.32 x 10^2^ ± 0.6 x 10	JSF3 1.0 x10^6^ ± 1.5 x10^5^ JSF4 6.6 x10^8^ ± 1.2 x10^8^ JSF7 5.9 x 10^8^ ± 3.2 x10^7^	0.43 ± 0.04	0.11 ± 0.01
MO1220 (O139)	JSF3, JSF4, JSF7	3.4 x 10 ± 0.4 x 10	2.7 x 10 ± 0.4 x 10	JSF3 5.6 x10^8^ ± 1.1 x10^8^ JSF4 1.1 x10^7^ ± 1.1 x10^6^ JSF7 1.0 x10^7^ ± 1.5 x10^6^	0.47 ± 0.05	0.23 ± 0.04
Mixed biofilm of C6706 (O1, El Tor) and MO1220 (O139)	JSF3, JSF4, JSF7	C6706 3.6 x10 ± 0.1 x10 MO1220 4.5x10 ±0.4x10	C6706 2.9 x10 ± 0.8 x10 MO1220 3.2x10±0.4 x10	JSF3 6.3 x10^8^ ± 1.1 x10^8^ JSF4 6.9 x10^8^ ± 1.2 x10^8^ JSF7 6.2 x10^8^± 4.6 x10^7^	0.46 ± 0.05	0.12 ± 0.02

The initial titer of different phages used were 1.5 x 10^7^ pfu/ml. The El Tor biotype *V*. *cholerae* O1 strain C6707 was susceptible to both JSF4 and JSF7, whereas the *V*. *cholerae* O139 strain was only susceptible to JSF3. Phage JSF7 could disperse biofilms of both these *V*. *cholerae* strains. Figures represent mean values and standard deviations of three independent observations.

*For JSF7 phage and C6706 derived biofilms, the observed increase in ratio of C6706 cells in the aqueous phase with and without phage was significantly lower compared to that for JSF7 phage and biofilms of strain MO1220 ((p = 0.0034).

### Genomics of JSF3, JSF4 and JSF7 phages

**Fig 4:**
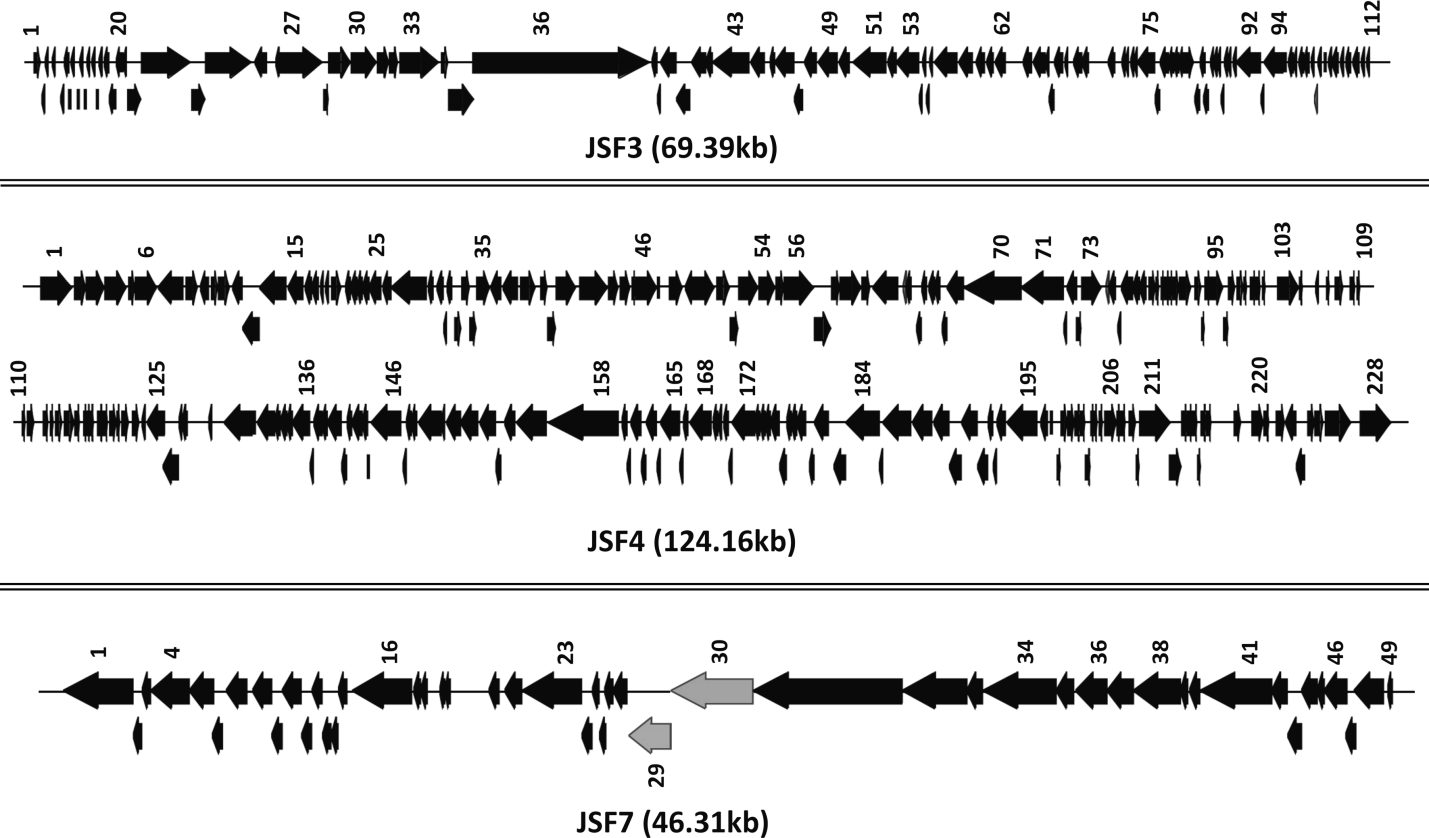
Schematic representation of the structure, size and direction of transcription of the open reading frames (ORFs) of phage JSF3, JSF4, and JSF7. The two ORFs designated ORF29 and ORF30 which are predicted to encode GDSL-like Lipase and Polysaccharide (Cycloalternan/pectin)-degrading enzymes respectively in JSF7 phage are shown with grey arrows.

Determination of the genomic sequence of JSF3, JSF4 and JSF7 phages revealed that these phage genomes comprise 69.39Kbp 124.16Kbp and 46.31Kbp nucleotides respectively. JSF3 contains 112 open reading frames whereas JSF4 and JSF7 comprised of 228 and 49 ORFs respectively. The genomic sequence of JSF3 was found to be 99% similar with that of a previously reported vibriophage JA-1 [18] whereas JSF4 phage has more than 98% sequence homology with a previously reported phage ICP1 [20]. However, sequence of JSF7 phage did not show any significant homology with any previously reported phage genome. The GC content of JSF7 genome was found to be 48% which is considerably higher than that of JSF3 (34%) and JSF4 (37%). Remarkably, the genomic sequence of JSF7 carried two ORFs which are predicted to encode GDSL-like Lipase and Polysaccharide (Cycloalternan/pectin)-degrading enzymes respectively. These enzymes were most likely responsible for the observed bio-film degrading function of JSF7 phage. However, further studies including mutational analysis and demonstration of concomitant lack of function will be necessary to confirm the presumed activity of these genes. The general structures of the genomes of JSF3, JSF4 and JSF7 phages with size and directions of the ORFs are presented in Fig 4. Sequences of JSF3, JSF4 and JSF7 phages are available under GenBank accession numbers KY065148; KY065147; and KY065149 respectively.

## Discussion

The results of this study have relevance to various aspects of our knowledge of microbial ecology, as well as potential application of phages to treat bacterial contamination in water. The aquatic environment in a cholera-endemic area is known to harbor a variety of phages that grow on *V*. *cholerae* with varying specificity towards different serogroups and strains (13). Previous studies suggested that these phages generally influence the *V*. *cholerae* population through their predatory role, while the bacteria are also known to survive phage attack through various means including restriction modification systems, mutations, modulation of receptors, and formation of biofilms [20, 21, 22, 23]. In view of reports implicating biofilms as a significant state of bacterial persistence in water and a vehicle for delivery of high dose of pathogenic *V*. *cholerae* there is a growing need for effective treatments of bacteria present in biofilms [3, 6, 8, 9]. To address this issue, we attempted to isolate phages which degrade biofilms, as well as phages which are active on planktonic *V*. *cholerae* cells in order to simultaneously attack biofilm matrices composed of extracellular polymeric substances, in addition to bacterial cells which are dispersed from biofilms.

The JSF7 phage characterized in this study was found to be active on biofilms of both phage-susceptible and resistant *V*. *cholerae* cells, suggesting that the phage did not require to infect the cells, but presumably carried enzymes that could act on biofilms extracellularly. In agreement with this assumption, genomic sequence of JSF7 revealed the presence of genes predicted to encode enzymes potentially capable of degrading biofilms. Bacterial cells released from biofilms of strain C6706 were susceptible to JSF7, and hence to some extent were killed by the phage. However, cells released from biofilms of the *V*. *cholerae* O139 strain were resistant to JSF7 phage and were hence not killed by JSF7. Thus, instead of reducing the concentration of *V*. *cholerae* in water, remarkably the action of JSF7 on *V*. *cholerae* O139 strain increased the concentration of planktonic *V*. *cholerae* cells in water, and hence addition of a second phage JSF3 was required to eliminate the *V*. *cholerae* O139 cells (Table 2).

Bacteria in biofilms are assumed to remain somewhat unaffected by phages unless the phages are capable of degrading the bioilm matrix. Predictably, biofilm associated *V*. *cholerae* cells were found to be mostly resistant to phages JSF3 and JSF4 as compared to the corresponding planktonic bacteria (Fig 3). We presume that the resistance was either due to a lack of accessibility of phages to the receptors on the bacterial surface or because biofilm associated cells are metabolically less active and hence may not support optimum phage-multiplication. However, cells dispersed from biofilms using the biofilm degrading activity of JSF7 phage were mostly susceptible to subsequent predation by their lytic phages JSF3 or JSF4 (Table 2). Thus, JSF7 not only dispersed cells from the biofilm matrices, but possible enzymatic degradation of the exopolysaccharide matrix by JSF7 might have stimulated the liberated cells to become rapidly active, and hence susceptible to the lytic phages. Furthermore, our results showed that effective elimination of both biofilm-associated and planktonic bacteria in water could be achieved by using a mixture of phages simultaneously, and at least one of these phages should be capable of dispersing biofilms.

Toxigenic strains of *V*. *cholerae* belonging to either O1 or O139 serogroups are capable of causing rapidly spreading epidemics of cholera. Although *V*. *cholerae* O139 is not isolated frequently in recent time, the existence of this serogroup in some environmental niche can not be ruled out. Notably, after the epidemics of 1992–1993 and a subsequent transient disappearance of the O139 strain there was a resurgence of the O139 strain in Bangladesh in 2002, causing another large epidemic [24]. Therefore, in this study, we included phages active against *V*. *cholerae* O1 as well as a phage that could kill *V*. *cholerae* O139. Moreover the inclusion of the O139 strain in this study demonstrated a scenario when biofilm degrading phages act on biofilms of phage-resistant strains. Under such circumstances, the activity of the phage essentially increases the concentration of active pathogenic bacteria in water instead of decreasing it, as commonly expected.

We further demonstrated that for effective removal of *V*. *cholerae* using phages it is essential to use a cocktail of multiple phage strains. The application of phages to control bacterial contamination has been limited mainly by the lack of sufficient variety of well characterized phages and unavailability of standardized protocols for using phages efficiently. The rapidly emerging resistance of bacteria to available antimicrobials have caused a renewed interest in testing the utility of phages to eliminate contamination of foodstuff or infection with pathogenic bacteria. However, bacterial contaminants often persist as biofilms which are somewhat resistant to phages. For efficient use of phages to control bacterial contamination, there is a need to identify and characterize phages capable of degrading biofilms. We predict that our results are likely to contribute towards developing improved methods for phage mediated treatment of water or foodstuff contaminated with bacterial biofilms.

Besides developing potential application for phage-mediated control of *V*. *cholerae*, our results may also contribute to an improved understanding of the role of phages in modulating the prevalence of pathogenic *V*. *cholerae* in water. Numerous reports suggest the involvement of environmental factors in triggering cholera epidemics [5,7,8,9] but the scope and mechanism of action of these diverse factors are yet to be fully elucidated. Phages in the environment have been found to influence the abundance of pathogenic *V*. *cholerae* in water samples and the incidence rates of cholera [8, 9, 25, 26]. Phages also play a role in emergence of pathogenic clones, and may also be involved in territorialism between different strains of *V*. *cholerae* [8, 13]. These reports suggest that phages in the environment likely influence the temporal dynamics of cholera epidemics, through their predatory effects on *V*. *cholerae* that reduces the abundance of the pathogen. Contrary to these notions, the results of the present study suggest that phages such as JSF7 may also contribute to raise the concentration of *V*. *cholerae* in water by dispersing biofilm-associated bacteria. Although in this study we have characterized the phage JSF7 as the only phage exhibiting biofilm-degrading activity, we identified this phage by screening merely 36 phage isolates (2.7%) in our sampling. Considering the immense number of phages which exist in the aquatic environment, it seems highly likely that the occurrence of biofilm-degrading phages is more common, and their collective effects may be significant. Moreover, the ability to degrade biofilms allows this group of phages to effectively access the bacterial host for reproduction, and hence biofilm-degrading property is likely to enhance their evolutionary fitness.

A variety of other factors also contribute and control the prevalence of *V*. *cholerae* and the occurrence of cholera outbreaks. For example, bacterial cell density-dependent gene expression termed “quorum sensing” which is regulated by signal molecules called autoinducers (AIs) also causes dispersion of biofilms [27, 28]. AIs also resuscitate dormant environmental cells of *V*. *cholerae* into actively dividing cells [29]. Interestingly, we also showed recently that AIs enhance resistance of *V*. *cholerae* to phages [23]. Enhanced infectivity of *V*. *cholerae* during a cholera epidemic has been suggested to be a result of inherent "hyperinfectivity" of cells shed in stools of cholera victims as well as the presence of biofilm-like clumps of cells which allows the delivery of the pathogen at a high dose to be able to infect a potential victim [3, 30]. On the other hand, lytic phages have been suggested to reduce the infectivity of *V*. *cholerae* by modulating the required infectious dose [9, 31]. In contrast, results of this study suggest that the JSF7 phage can also cause dispersion of biofilms, leading to abundance and spread of actively dividing *V*. *cholerae* cells in water, which is a risk factor for the occurrence of cholera outbreaks [7]. Thus a combination of multiple factors fine tune the prevalence and infectivity of *V*. *cholerae* in water, of which phages are important agents both in deceasing as well as increasing the prevalence of the pathogen. In summary, besides possible application of a mixture of phages to treat water contaminated with *V*. *cholerae* to reduce transmission, the results of this study provide interesting refinements to our understanding of the potential role of phages in the ecology and epidemiology of cholera.

## Supporting information

S1 Table Susceptibility pattern of various bacteria to JSF3, JSF4 and JSF7 phages. (DOCX)

S1 Fig Stability of JSF3, JSF4 and JSF7 phages at different temperature, pH and salinity. (TIF)
