# Supplementary material for: Environmental bacteriophages active on biofilms and planktonic forms of toxigenic Vibrio cholerae: Potential relevance in cholera epidemiology
Source: PLoS One. 2017 Jul 10;12(7):e0180838. doi: 10.1371/journal.pone.0180838 (PMC5507324; doi:10.1371/journal.pone.0180838)
Supplement: S1 Table — (DOCX) [file pone.0180838.s001.docx]

**Table S1**. Susceptibility pattern of various bacteria to phages JSF3, JSF4 and JSF7

| Organism | No. of isolates | Source of isolates | No. of isolates susceptible to different phages | | |
| --- | --- | --- | --- | --- | --- |
|  |  |  | JSF-3 | JSF-4 | JSF-7 |
| *V. cholerae* O1; Classical Biotype | 17 | Patient and Environment | 0 | 3 | 10 |
| *V. cholerae* O1; El Tor Biotype | 18 | Patient and Environment | 0 | 12 | 7 |
| *V. cholerae* O139 | 12 | Patient and Environment | 12 | 0 | 0 |
| *V. cholerae* non-O1 non-O139 | 9 | Environment | 0 | 0 | 0 |
|  |  |  |  |  |  |
| *Shigella dysenteriae* 1 | 11 | Patient | 0 | 0 | 0 |
| *S. flexneri* | 18 | Patient | 0 | 0 | 0 |
| S. boydie | 11 | Patient | 0 | 0 | 0 |
| S. sonnie | 3 | Patient | 0 | 0 | 0 |
|  |  |  |  |  |  |
| Enterotoxigenic  *E. coli* (ETEC) | 20 | Patient | 0 | 0 | 0 |
| Enteropathogenic  *E. coli* (EPEC) | 18 | Patient | 0 | 0 | 0 |
| *Providencia alcalifaciens* | 3 | Patient and environmental | 0 | 0 | 0 |
| *V. parahemolyticus* | 1 | patient | 0 | 0 | 0 |
| *Escherichi.coli* HB101 | 1 | Laboratory collection | 0 | 0 | 0 |
