# Supplementary figures and images for: Environmental bacteriophages active on biofilms and planktonic forms of toxigenic Vibrio cholerae: Potential relevance in cholera epidemiology
Source: PLoS One. 2017 Jul 10;12(7):e0180838. doi: 10.1371/journal.pone.0180838 (PMC5507324; doi:10.1371/journal.pone.0180838)

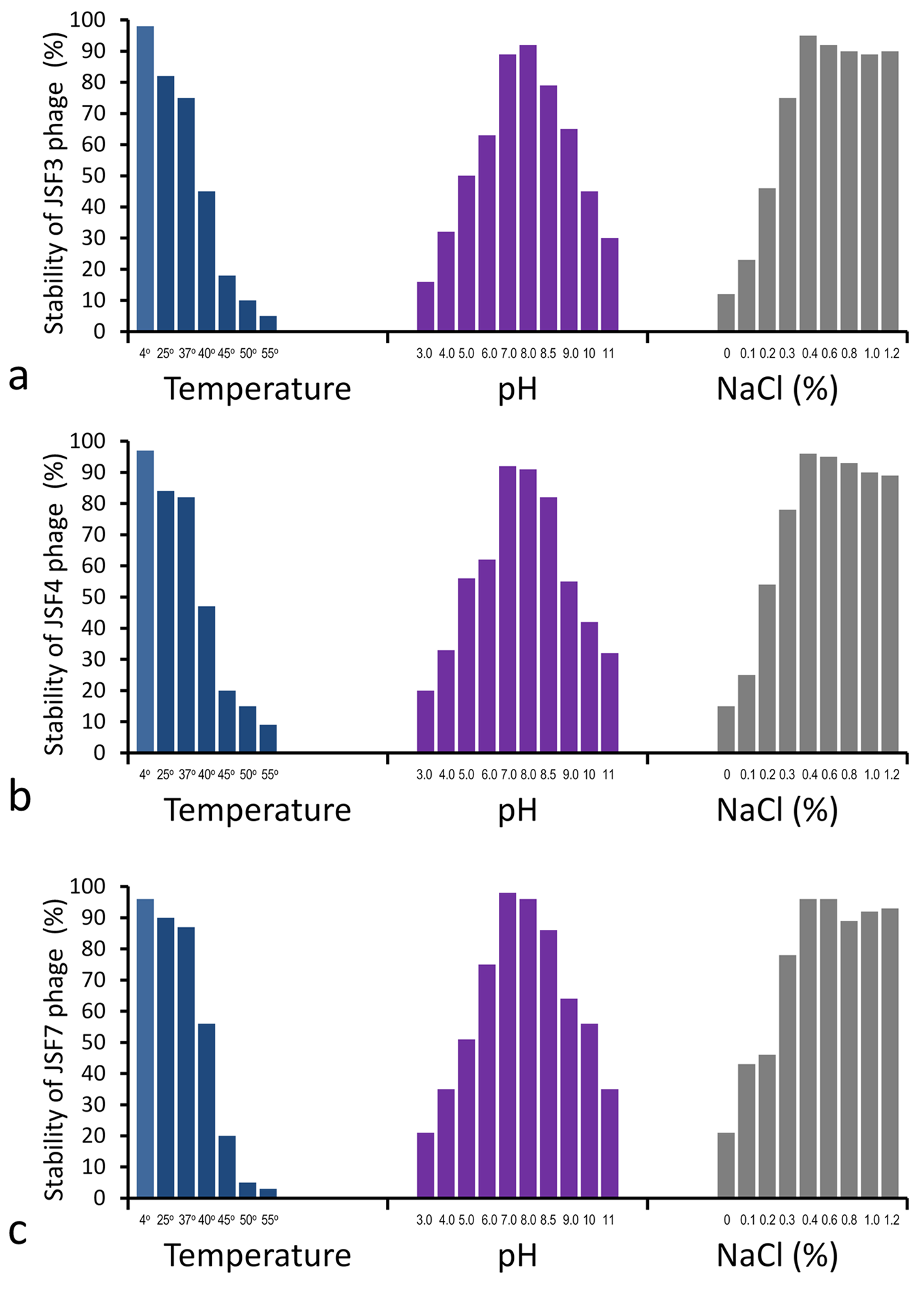

Supplement: S1 Fig — (TIF) [file pone.0180838.s002.TIF]
